# Identification of an AP2-family Protein That Is Critical for Malaria Liver Stage Development

**DOI:** 10.1371/journal.pone.0047557

**Published:** 2012-11-07

**Authors:** Shiroh Iwanaga, Izumi Kaneko, Tomomi Kato, Masao Yuda

**Affiliations:** Department of Medical Zoology, Mie University School of Medicine, Tsu, Mie, Japan; Museum National d’Histoire Naturelle, France

## Abstract

Liver-stage malaria parasites are a promising target for drugs and vaccines against malaria infection. However, little is currently known about gene regulation in this stage. In this study, we used the rodent malaria parasite *Plasmodium berghei* and showed that an AP2-family transcription factor, designated AP2-L, plays a critical role in the liver-stage development of the parasite. *AP2-L*-depleted parasites proliferated normally in blood and in mosquitoes. However, the ability of these parasites to infect the liver was approximately 10,000 times lower than that of wild-type parasites. In vitro assays showed that the sporozoites of these parasites invaded hepatocytes normally but that their development stopped in the middle of the liver schizont stage. Expression profiling using transgenic *P. berghei* showed that fluorescent protein-tagged AP2-L increased rapidly during the liver schizont stage but suddenly disappeared with the formation of the mature liver schizont. DNA microarray analysis showed that the expression of several genes, including those of parasitophorous vacuole membrane proteins, was significantly decreased in the early liver stage of *AP2-L*-depleted parasites. Investigation of the targets of this transcription factor should greatly promote the exploration of liver-stage antigens and the elucidation of the mechanisms of hepatocyte infection by malaria parasites.

## Introduction

The transmission of malaria to humans is initiated by the bite of an *Anopheles* mosquito, which deposits malaria sporozoites from the mosquito salivary glands into the skin of the host. The sporozoites then migrate to the liver, invade hepatocytes and transform into liver-stage (LS) parasites inside a parasitophorous vacuole (PV). The parasites undergo nuclear divisions and finally multiply into thousands of merozoites, which infect erythrocytes.

The LS parasite is one of the most attractive targets for malaria vaccine development. Robust and long-term protection against malaria transmission has been induced in mice and humans by infecting them with sporozoites that are attenuated by irradiation or genetic modifications [1]–[3]. Development of these parasites is arrested in the LS, and cellular immunity against LS parasites is thought to have an important role in this protection [4]–[6]. However, few antigens of this parasite, which are specific for this stage, have been identified.

To explore candidate antigens in LS parasites and to elucidate the mechanisms of liver infection, transcriptomic and proteomic studies have been carried out [7], but a comprehensive understanding of gene expression has not been achieved. The lack of progress in this area results from the difficulty of collecting sufficient quantities of LS parasites for expression analyses and genetic studies. Moreover, this difficulty is greatly increased in the early stage because of the small size of the parasite. The mechanisms of gene regulation in the LS also remain largely unknown. In particular, the transcription factors that control stage-specific gene expression have not been identified.

Apetala 2 (AP2)-family proteins are transcription factors that have DNA-binding domains of ∼60 amino acids called AP2 domains. Recently, AP2 genes have been found in the genomes of *Plasmodium* parasites [8]–[10]. In *Plasmodium falciparum*, the most important human malaria parasite, 27 AP2-family genes have been identified [9]. Among these genes, 26 are conserved in the other *Plasmodium* species whose entire genomes have been sequenced. Each member of this family has 1 to 4 AP2 domains, and the amino acid sequences of these domains are highly conserved among *Plasmodium* orthologs. At present, the AP2 family is the only family of sequence-specific transcription factors whose functions have been demonstrated in *Plasmodium* species. The extraordinarily small number of sequence-specific transcription factors identified in the *Plasmodium* genome suggests that AP2-family proteins have central roles in gene regulation in these parasites and that *Plasmodium* parasites have a unique system of gene regulation to maintain their complex life cycle.

AP2-family genes are expressed in the asexual blood stages of the *Plasmodium* life cycle [8]. AP2-family transcription factors play central roles in gene expression in ookinetes and sporozoites [11], [12]. In this study, we report that an AP2-family transcription factor of the rodent malaria parasite *P. berghei* (PlasmoDB ID, PBANKA_021440, designated *AP2-L*) is expressed in LS parasites. We show that the disruption of *AP2-L* almost completely arrests LS development without affecting the proliferation of other stages in the life cycle.

## Results and Discussion

### *AP2-L* is Expressed in Sporozoites and is Necessary for Parasite Infection of the Liver

**Figure 1:**
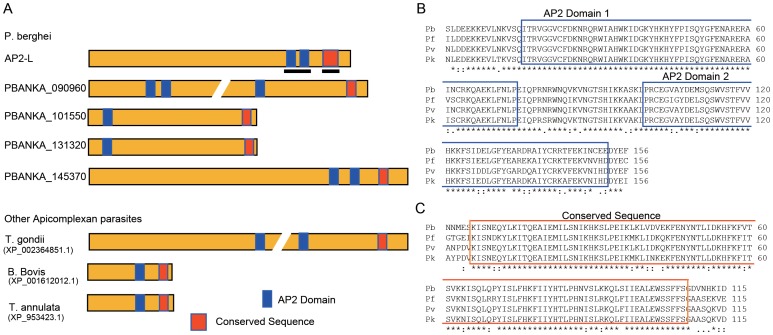
AP2-L and related proteins in apicomplexan parasites. A. Schematic diagrams of *P. berghei* AP2-L and related proteins are shown. The paralogs in *P. berghei* are indicated by their gene identifiers in PlasmoDB. The related proteins in other apicomplexan parasites are indicated by the species name and their GenBank accession number. AP2 domains (blue) and another conserved sequence (red) are highlighted by rectangles. Bars indicate the regions shown in B and C. B. Amino acid sequences of a region containing two A domains (highlighted with rectangles) are compared in the AP2-Ls of four *Plasmodium* species: *P. berghei* (Pb), *P. falciparum* (Pf), *P. vivax* (Pv), and *P. knowlesi* (Pk). The amino acids conserved across all four species are indicated by asterisks. This region is indicated in A by the left bar. C. Amino acid sequences of another region conserved among the AP2-Ls. This region is indicated in A by the right bar. The amino acids conserved across all four species are indicated by asterisks.

To investigate the involvement of AP2-family transcription factors in liver infection by *P. berghei*, we searched for expressed sequence tags (ESTs) encoding AP2 transcription factors in our *P. berghei* sporozoite EST database (available in PlasmoDB, http://PlasmoDB.org). We found several ESTs encoding the AP2-family gene PBANKA_021440 (hereafter *AP2-L*). *AP2-L* encodes a protein of 1272 amino acids with two AP2 domains (Fig. 1A). These domains are located near the C-terminus of the protein and are separated from each other by a short linker. *AP2-L* orthologs are present in other *Plasmodium* species, including *P. falciparum,* and the amino acid sequences of the two AP2 domains, including the linker region, are highly conserved (Fig. 1B). The comparison of the amino acid sequences of these orthologs revealed another highly conserved region of approximately 100 amino acids C-terminal to the AP2 domains (Figs. 1A and 1C). The overall amino acid sequence identity of this region between *P. berghei* and *P falciparum* proteins is 84%. Known functional motifs were not found in this region by a search using InterProScan (http://www.ebi.ac.uk/InterProScan). BLAST searches of public databases using this sequence showed that related sequences exist for some *Plasmodium* AP2-family proteins (Figures 1A and S1A) and in AP2-family proteins of other apicomplexan parasites (Figures 1A and S1B). These sequences are all located near the C-terminus (Fig. 1A), suggesting that they have a common function and that these proteins constitute a subfamily of apicomplexan AP2-family proteins.

**Figure 2:**
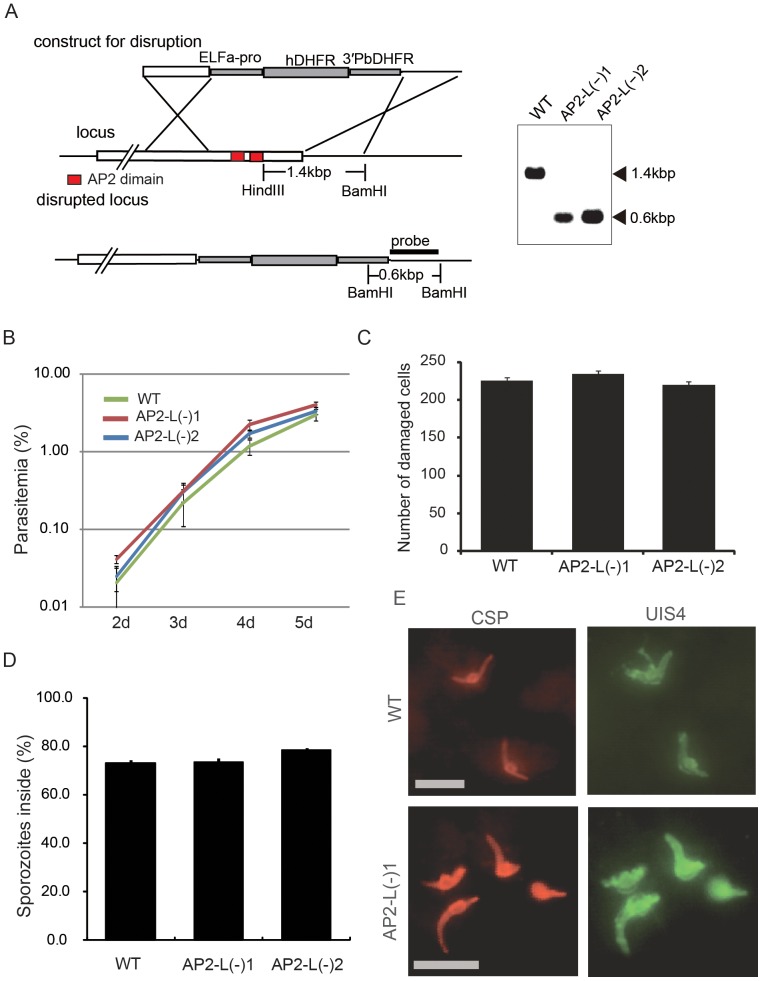
Deletion of the AP2-L gene does not affect the ability of sporozoites to invade hepatocytes. A. Schematic diagram of targeting *P. berghei AP2-L* by double cross-over homologous recombination. A Southern blot analysis of wild-type (WT) and two independent *AP2-L* knockout populations (*AP2-L*(−) 1 and *AP2-L*(−) 2) is shown on the right. B. Replication of asexual blood stages in mice. Mice were intravenously injected with blood infected with *AP2-L*(−) or wild-type (WT) parasites (∼1×10^5^ parasites per mouse). The parasitemia of each mouse was checked each day. The values are the mean ± standard error of the mean (SEM) for four measurements. C. Cell-traversal assay in HepG2 cells. Sporozoites (4×10^4^) were inoculated into a culture of HepG2 cells and incubated at 37°C for 30 min. The number of injured cells that incorporated FITC-labeled dextran was counted. The values are the mean ± SEM of four measurements. D. Double staining assay. Sporozoites (5×10^4^) were inoculated into a culture of HepG2 cells and incubated at 37°C for 1 h. The sporozoites outside (stained both red and green) and inside (stained only red) the HepG2 cells were counted independently. The results are shown as the percentage of sporozoites inside the cell out of the total. The values are the mean ± SEM of eight measurements. E. Fluorescence images of *AP2-L*(−) 1 and wild-type parasites inside HepG2 cells 6 h after sporozoite inoculation. The parasites were stained with anti-CSP (red) and anti-UIS4 (green) antibodies. The scale bars represent 10 µm.

To investigate the function of *AP2-L* in the parasite life cycle, we performed targeted knockout experiments in *P. berghei.* The *AP2-L* gene was knocked out by the insertion of a targeting construct containing the pyrimethamine-resistance human dihydrofolate reductase (DHFR) gene into the *AP2-L* locus by double cross-over recombination (Figure 2A). Parasites that integrated the construct were separated from wild-type parasites by limiting dilution. Two parasite populations prepared by independent transfections were used in the following experiments.

*AP2-L*(−) parasites had an intra-erythrocytic replication rate nearly identical to that of wild-type parasites (Figure 2B). All mosquito stages (oocysts, oocyst sporozoites and salivary gland sporozoites) formed in numbers similar to those of wild-type parasites (Table S1). The motility rates of salivary gland *AP2-L*(−) sporozoites were also normal (>90%). To examine liver infectivity, salivary gland sporozoites were injected intravenously into rats, and the prepatent time for parasitemia was monitored (Table 1). When inoculated with 30,000 sporozoites, 15 out of 16 rats were infected, and the prepatent time for parasitemia in the infected rats was significantly extended (average 4.5–5.3 days) as compared with the rats inoculated with wild-type sporozoites. From the replication rate of the asexual blood stage (∼6–10 fold per day) and the delay in parasitemia, the liver infection ability of *AP2-L*(−) parasites was estimated to be approximately 10,000 times lower than that of wild-type parasites.

**Table 1:** The ability of *AP2-L*(−) parasites to infect the liver is greatly reduced.

	Parasite	Number of injected sporozoites^a^	Number of infected rats/injected	Prepatent time (days)^b^
Experiment 1	Wild-type	30,000	4/4	3.0
	*AP2-L*(−) 1	30,000	3/4	7.5
	*AP2-L*(−) 2	30,000	4/4	7.5
Experiment 2	Wild-type	30,000	4/4	3.0
	*AP2-L*(−) 1	30,000	4/4	7.8
	*AP2-L*(−) 2	30,000	4/4	8.3

a. Salivary gland sporozoites were collected 24 days after an infective blood meal.

b. The average prepatent time for infected rats. Parasitemia was checked at one-day intervals starting 2 days after the challenge.

### *AP2-L*(−) Parasites Migrate through Cells Normally and Productively Invade Hepatocytes

The reduced ability of *AP2-L*(−) parasites to infect liver could be due to one of the following possibilities: (1) the cell-traversal ability of *AP2-L*(−) sporozoites is impaired, and thus, they do not arrive at the liver parenchyma; (2) *AP2-L*(−) sporozoites arrive at hepatocytes but cannot invade them productively (i.e., by forming a PV); (3) *AP2-L*(−) sporozoites invade hepatocytes normally but are unable to develop into merozoites. First, we examined the first and second possibilities with in vitro assays using salivary gland sporozoites.

The cell-traversal ability of sporozoites was determined by cell-injury assays in HepG2 cells, a human hepatoma-derived cell line. Sporozoites were added to a culture of HepG2 cells, and injured cells, which were marked by the incorporation of fluorescein isothiocyanate (FITC)-labeled dextran, were counted under a fluorescence microscope. Similar numbers of injured cells were found upon infection with both *AP2-L*(−) and wild-type parasites (Figure 2C), indicating that *AP2-L*(−) sporozoites can rupture and traverse cells normally.

Next, double staining assays were performed for examining the ability of *AP2-L*(−) sporozoites to productively invade hepatocytes; this ability can be indirectly estimated because the proportion of parasites inside the hepatocytes decreases significantly when the parasites cannot invade the hepatocytes [13]. Sporozoites were added to a culture of HepG2 cells, allowed to invade for 30 min, and differentially stained with anti-circumsporozoite protein (CSP) antibodies according to their location, i.e., outside or inside the HepG2 cells (Figure 2D). Approximately the same proportion of sporozoites (70–80%) was observed to be inside the HepG2 cells whether they were wild type or *AP2-L*(−), suggesting that the sporozoites had retained their normal ability to invade the hepatocytes productively. We further checked whether *AP2-L*(−) sporozoites form an intact PV membrane inside HepG2 cells by immunofluorescence staining with antibodies against the protein upregulated in infective sporozoites 4 (UIS4) [14], which is observed in the PV membrane after sporozoite invasion. Sporozoites were inoculated into a culture of HepG2 cells and immunofluorescently stained at 6 hours post inoculation (hpi) with antibodies against UIS4 and CSP. *AP2-L*(−) sporozoites had already started transforming into the LS, as had wild-type parasites (Figure 2E). Staining with antibodies against UIS4 showed that the majority of *AP2-L*(−) sporozoites (∼80%) had formed a PV membrane. These results indicate that the sporozoites of *AP2-L*(−) parasites retain their normal ability to migrate into the liver and productively invade hepatocytes.

### *AP2-L* is Necessary for Parasite Development in Hepatocytes

**Figure 3:**
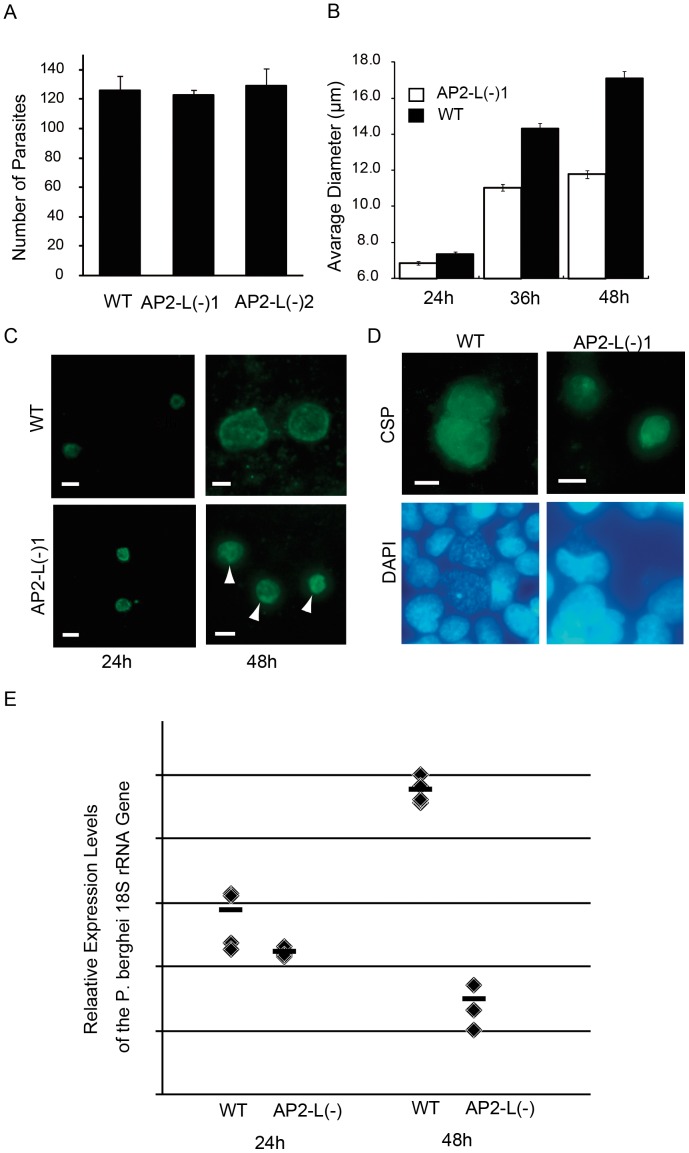
LS development is arrested during the schizont stage in *AP2-L*(−) parasites. A. Sporozoites (2×10^4^) were inoculated into a culture of HepG2 cells and incubated at 37°C. The number of LS parasites was counted 48 hpi after staining with anti-CSP antibody. The data are the mean ± SEM of four measurements. B. The diameters of LS *AP2-L*(−) 1 and wild-type parasites were compared. When the parasites were not round, the average of the longest and the shortest axis was used as the diameter. The horizontal axis indicates the number of hours post-sporozoite inoculation. The data are the mean ± SEM of 200 measurements. C. Fluorescence images of *AP2-L*(−) 1 and wild-type (WT) LS parasites at 24 hpi and 48 hpi. The parasites were stained with an anti-CSP antibody. The arrows indicate densely stained portions that were specifically observed in *AP2-L*(−) LS parasites. D. Representative image of wild-type and *AP2L*(−) 1 LS parasites at 53 hpi with nuclear staining by DAPI. The scale bars in C and D represent 10 µm. E. Parasite burden in the rat liver was compared between the wild-type and *AP2-L*(−) parasites. Wild-type or *AP2-L*(−) salivary gland sporozoites were intravenously injected into rats, and the liver was dissected after perfusion at 24 hpi or 48 hpi. Parasite loads in the liver were determined by measuring *P. berghei* 18S rRNA using RT-PCR. The rat glyceraldehyde 3-phosphate dehydrogenase gene was used as an internal control to normalize the mean cycle threshold value of the rat 18S rRNA gene. Four rats were used for each condition.

The delay in patency, despite normal sporozoite traversal and invasion, suggests that *AP2-L*(−) parasites undergo defective LS development. We next investigated the development of the *AP2-L*(−) LS parasites by in vitro culturing using HepG2 cells. First, cultured LS parasites were immunofluorescently stained with an anti-CSP antibody at 48 hpi. At this time, the number of LS parasites formed in HepG2 cells was similar between wild-type and *AP2-L*(−) parasites (Figure 3A), but the *AP2-L*(−) LS parasites were obviously smaller than the wild-type LS parasites (data not shown, see also Figure 3C). We therefore checked the development of LS parasites at different time points (24 hpi, 36 hpi, and 48 hpi, Figure 3B and Figure 3C). At 24 hpi, the difference in size between the wild-type and *AP2-L*(−) parasites was still subtle, but it became clear at 36 hpi. After 36 hpi, the sizes of the *AP2-L*(−) LS parasites scarcely increased, whereas the wild-type parasites continued to grow until 48 hpi. In accordance with the growth of the LS parasites, arrest of nuclear division became clear at 36 hpi in the *AP2-L*(−) parasites. The appearance of the nuclei remained the same, and no *AP2-L*(−) LS parasites in the cytomere stage were formed in the later stage (Figure 3D). Furthermore, the GFP signals of the *AP2-L*(−) parasites started to show heterogeneous distributions at 36 hpi, suggesting that degenerative changes had occurred (Figure 3C and 3D). These observations indicate that the development of *AP2-L*(−) parasites almost completely arrested at about 36 hpi, i.e., in the middle of the schizont stage, which caused a severe decrease in their ability to infect the liver (Table 1).

Further, we compared parasite loads in the rat liver between wild-type and *AP2-L*(−) parasites (Figure 3E). After intravenous inoculation of salivary gland sporozoites, rats were dissected at 24 hpi or 48 hpi, and parasite loads in the liver were determined using real time reverse-transcription PCR (RT-PCR) as the ratio of the parasite 18S rRNA to rat glyceraldehyde 3-phosphate dehydrogenase control. At 24 hpi, parasite loads in the rats inoculated with *AP2-L*(−) parasites were the same or lower than those in rats inoculated with the wild-type parasites. Subsequently, the parasite loads decreased by approximately sixfold in rats inoculated with *AP2-L*(−) parasites until 48 hpi, while an approximately 100-fold increase was observed in the rats inoculated with wild-type parasites. As a result, differences between the *AP2-L*(−) and wild-type parasite loads increased to more than 1000-fold at 48 hpi, coinciding well with values estimated from the delay in rat parasitemia (Table 1). These results clearly demonstrate that parasite development in the liver is hindered by the disruption of *AP2-L* and that decreased liver infection capacity in *AP2-L*(−) parasites can be attributed to their impaired LS development.

### *AP2-L* is Localized in the Nucleus of Erythrocytic Trophozoites, Sporozoites and LS Parasites

**Figure 4:**
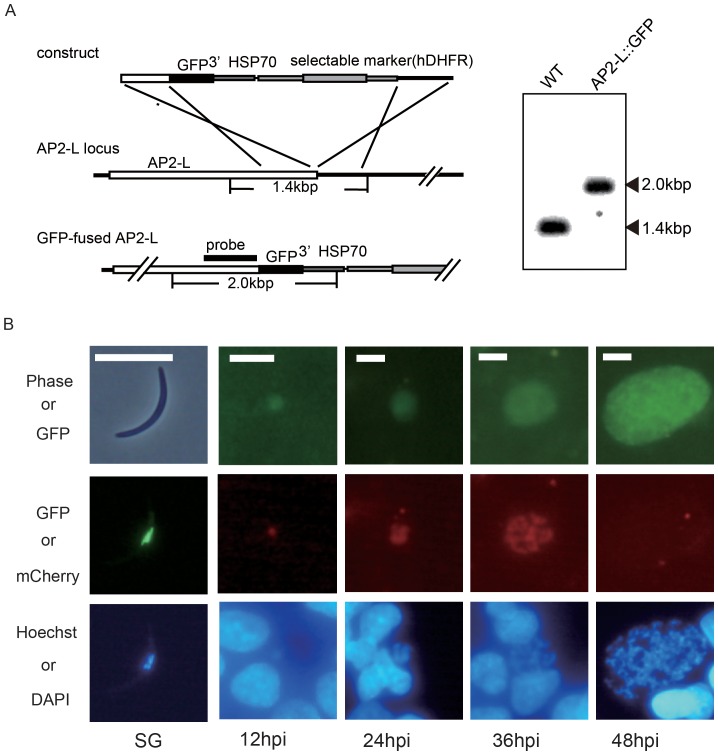
The expression profile of *AP2-L* in *P. berghei* pre-erythrocytic stages. A. A schematic diagram showing the preparation of transgenic *P. berghei* expressing GFP-tagged AP2-L. The expression construct was introduced into the 3′ portion of the endogenous *AP2-L* gene by double cross-over homologous recombination. The results of the Southern blot analysis of wild-type (WT) and transgenic *AP2-L* parasites (*AP2-L ::GFP* (−)) are shown on the left. B. Fluorescence images of a salivary gland sporozoite (SG; expressing GFP-tagged AP2-L, extreme left column) and LS parasites (expressing mCherry-tagged AP2-L, other columns). LS parasites were harvested at 12-h intervals following sporozoite inoculation. The LS parasites expressed GFP constitutively in the cytoplasm (upper images). The nuclei were stained with Hoechst 21486 (in sporozoites) or DAPI (in LS parasites). The scale bars represent 10 µm.

The expression profile of *AP2-L* in the blood and mosquito stages was investigated using transgenic *P. berghei* parasites expressing green fluorescent protein (GFP)-fused *AP2-L* (*AP2-L::GFP* parasites) (Figure 4A). The fusion of GFP did not affect parasite proliferation through the life cycle, including the LS (Table S2). During the blood stages, GFP-fused AP2-L was observed in the nuclei of trophozoites but not in the ring or schizont forms or the gametocytes (Figure S2). In the mosquito stages, GFP-fused AP2-L was first observed in the nuclei of the oocyst sporozoites (10 days after an infective blood meal, data not shown), and even more strongly in the nucleus of the salivary gland sporozoites (Figure 4B).

Expression of *AP2-L* in LS parasites was investigated by culturing in HepG2 cells. To identify LS parasites in the culture, we prepared parasites expressing mCherry (red fluorescent protein)-tagged AP2-L (*AP2-L::mCherry* parasites) from the *P. berghei* ANKA 507cl1 mutant line [15], which constitutively expresses GFP in the cytoplasm, essentially in the same manner as *AP2-L::GFP* parasites. Salivary gland sporozoites of these parasites exhibited normal infectivity in rats (Table S2). Expression profiles and the subcellular localization of AP2-L in the blood and mosquito stages of *AP2-L::mCherry* parasites were the same as in those of the *AP2-L::GFP* parasites (Figure S3). The expression of *AP2-L* in LS parasites was assessed at 12-h intervals after inoculation of *AP2-L::mCherry* sporozoites into a culture of HepG2 cells. mCherry-tagged AP2-L was visible in the nucleus beginning at 12 hpi and rapidly increased from 24 hpi to 36 hpi with the progress of nuclear division. However, fluorescent signals completely disappeared in the large sized LS parasites at 48 hpi (Figures 4B and S4), suggesting that AP2-L is rapidly eliminated from the LS parasites when parasites complete schizogony. The sudden and complete disappearance of AP2-L during this period indicates that other transcription factors begin to regulate gene expression in the LS parasites by replacing AP2-L. Sequential expression of the AP2 transcription factors may occur during LS, as has been reported for the asexual blood stages [8].

The expression of *AP2-L* in the blood-stage trophozoites suggests that some target genes are commonly expressed between the asexual intra-erythrocytic stage and the LS. Expression of *AP2-L* in the asexual blood stages has also been reported for *P. falciparum,* in which the expression of *AP2-L* was observed in the ring forms by DNA microarray analysis [8]. However, it remains unclear why disruption of *AP2-L* had no effect on parasite proliferation in the blood stages, but severe impairment of schizogony occurred in the LS. Furthermore, the expression profile in the asexual blood stages was not identical to that in the LS; peak expression of *AP2-L* was observed during the schizont stage in the LS but during the trophozoite stage (i.e., before the schizont) in the blood stage. Therefore, we speculate that *AP2-L* is expressed in the intra-erythrocytic stage, but it may not function in this stage. Further investigation is necessary to elucidate the role of this transcription factor in the blood stage.

### Expression of *AP2-L* in Salivary Gland Sporozoites is Significantly Reduced in the *SLARP*(−) Parasites

Several genes involved in early LS development, such as *UIS3* and *UIS4* [2], [14], are transcribed in the sporozoite stage; however, their transcripts are silently stored until the subsequent liver stage. Sporozoite and LS asparagine-rich protein (SLARP) (also known as sporozoite asparagine-rich protein 1 [SAP1]) has been suggested to be a specific regulator of these genes [16], [17] because expression of these genes is significantly reduced in the *SLARP*-depleted (*SLARP*(−)) salivary gland sporozoites. Aly et al. reported that the reduction occurred because their transcripts, which are stabilized in stress granules in the cytoplasm of the salivary gland sporozoite, are routed towards degeneration pathways in the *SLARP*(−) sporozoites [17], [18]. We examined whether expression of *AP2-L* is also reduced in the *SLARP*(−) *P. berghei* salivary gland sporozoites using RT-PCR (Figures S5 and S6). Expression of *AP2-L* in the *SLARP*(−) salivary gland sporozoites was approximately 10 times lower than that in the wild-type parasites. This suggests that expression of *AP2-L* is controlled at the translational level. In contrast, as described above, analysis with the *AP2-L::GFP* parasites showed that GFP-tagged AP2-L was already visible in the nuclei of the salivary gland sporozoites (Figures 4B and S3). This result indicates that some of its transcripts are translated during the sporozoite stage. Translation of AP2-L in the sporozoite stage implies that the AP2-L protein is directly carried to the LS and induces its target genes before the transcripts of *AP2-L* are translated in the LS.

### The Expression of Several Genes is Decreased in the Early LS of *AP2-L*(−) Parasites

Next, we compared the gene expression in the LS between wild-type and *AP2-L*(−) parasites by DNA microarray analysis. For this study, a DNA microarray for *P. berghei* was newly designed based on the genome annotation published in PlasmoDB (http://plasmodb.org/plasmo/). This microarray contained probes for all annotated *P. berghei* genes (∼4800 genes). To minimize the secondary effects of *AP2-L* disruption on gene expression and obtain direct targets of this transcription factor, samples were obtained from LS parasites cultured for 24 hours in HepG2 cells, when only small differences in the sizes were observed between wild-type and *AP2-L*(−) LS parasites (see Figure 3B and 3C). Sporozoites (1×10^5^) were inoculated in each culture well of an 8-well chamber slide, and RNA was extracted from all cells in the well, that is, both uninfected and infected HepG2 cells. Because the samples used for the analysis should have contained only a small amount of parasite RNA, the obtained signals were expected to be weak. To increase the accuracy of the analysis, ten and seven biologically independent samples were used for wild-type and *AP2-L*(−) parasites, respectively, and genes whose expression decreased more than three-fold in *AP2-L*(−) parasites for all independent probes (four independent probes were attributed to each gene in this array) were selected as candidate target genes. A total of 11 genes were identified to meet these criteria (Table 2). These genes included five genes that are reportedly expressed in the LS (*UIS2*, *UIS3*, *UIS4*, *EXP1* and liver-specific protein 1 (*LISP1*)). Six of the 11 genes have a putative signal sequence (PBANKA_100300, PBANKA_144170, *UIS3*, *UIS4*, *EXP1* and *LISP1*), and four encode PV membrane proteins (*UIS3*, *UIS4*, *EXP1* and *LISP1*).

**Table 2:** Genes whose expression is decreased in *AP2-L*(−) parasites.

gene ID	fold decrease	Functional annotation	signal peptide
PBANKA_100300	41.49	sequestrin	+
PBANKA_102460	12.09	liver specific protein 1 (LISP1 )	+
PBANKA_041240	6.384	zinc finger protein, putative	−
PBANKA_050120	5.749	UIS4	+
PBANKA_020280	5.353	adenylate kinase	−
PBANKA_146260	5.35	conserved Plasmodium protein	−
PBANKA_132800	5.31	UIS2, serine/threonine protein phosphatase	−
PBANKA_144170	5.043	conserved Plasmodium protein	+
PBANKA_092670	4.545	EXP1	+
PBANKA_020830	3.73	transporter, putative	−
PBANKA_140080	3.561	UIS3	+

DNA microarray analysis was performed using LS cultures at 24 hpi. Genes whose expression was decreased at least three-fold by depletion of *AP2-L* are listed. Each gene is indicated by its PlasmoDB gene ID. The functional annotations are essentially in line with PlasmoDB. Signal sequences were predicted with SignalP (http://www.cbs.dtu.dk/services/SignalP/).

This analysis also showed that the expression of the heat shock protein 70 (HSP70) genes (PBANKA_081890, PBANKA_091440, and PBANKA_135720) scarcely changed (within 25% differences) between wild-type and *AP2-L*(−) parasites. We carried out RT-PCR analysis of *UIS3*, *UIS4*, and *LISP1* with PBANKA_091440 as an internal control. As shown in Figure S7, the expression of these three genes was significantly reduced in the *AP2-L*(−) parasites, confirming the results of the microarray analysis.

Because some of these genes (*UIS2*, *UIS3*, *UIS4*) are also expressed in salivary gland sporozoites, the expression of these genes may have been decreased prior to liver infection in the *AP2-L*(−) parasites. Therefore, we compared the gene expression between wild-type and *AP2-L*(−) parasites in the salivary gland sporozoites by DNA microarray analysis. The DNA microarray used in this analysis contained three independent probes for each *P. berghei* gene. Five biologically independent samples were prepared for each genotype, and the genes whose expression decreased more than threefold in *AP2-L*(−) parasites in all independent probes were selected as reduced genes. *AP2-L* was the only gene selected to meet these criteria among all *P. berghei* genes. Differences in the expression levels of five genes in Table 2 were within 50% between the two groups (*UIS2*, *UIS3*, *UIS4*, PBANKA_041240, and PBANKA_020830). The other six genes in Table 2 were filtered out as weakly expressed genes during the analysis process, indicating that they are not expressed in the sporozoite stage but are induced after liver infection. These results strongly suggest that AP2-L does not function in the sporozoite stage. We further examined the expression of *UIS3* and *UIS4* in salivary gland sporozoites by using RT-PCR. The CSP gene was used as an internal control to normalize the PCR data (the difference in the expression level of *CSP* in the DNA microarray analysis was less than 20% between wild-type and *AP2-L*(−) parasites). As shown in Figure S8, no significant differences were observed between the wild-type and *AP2-L*(−) salivary gland sporozoites. The normal expression of *UIS3* and *UIS4* in *AP2-L*(−) sporozoites is consistent with the finding that they were surrounded by normal PV membranes shortly after liver invasion (Figure 2E).

We next searched for the binding motif of AP2-L in the upstream regions of the candidate target genes using the same statistical method used in our previous papers [11], [12]. However, we could not obtain conclusive results from the analysis (data not shown). One possible reason for this failure is that the DNA binding domains of AP2-L recognize two independent sequences on the promoter simultaneously. Campbell et al. reported that neither single AP2 domain of *P. falciparum* AP2-L showed clear DNA binding properties [19]. This finding might indicate that these AP2 domains do not have sufficient binding affinities for DNA when separated from each other and thus a combination of two independent sequences is essential for determining binding specificity. Statistical analyses may not be suitable for the identification of such a complicated motif, and step-by-step approaches might be needed as the first step to address this issue, for example, to identify the location of each binding sequence by promoter assays using various deletion constructs.

Considering the results of our previous studies in other AP2-family transcription factors, AP2-L could have more target genes in LS parasites. We suspected that the 11 genes identified by the microarray analysis might cover only a portion of the target genes of AP2-L. However, the fact that half encode proteins with a signal sequence, and particularly that four encode PV membrane proteins, suggests that the target genes of AP2-L are strongly involved in host-parasite interactions in the LS. During schizont formation, the PV is rapidly enlarged because of the growth of the parasite. The PV membrane and PV membrane proteins support this growth by providing the parasite with an environment suitable for proliferation, for example, by taking up nutrients from their surroundings. Considering the importance of PV membrane proteins in this period, the reduced expression of these genes might be one of the causes of parasite growth arrest in the middle of the schizont stage. In addition to the PV membrane protein genes, the expression of two genes encoding putative secretory proteins (PBANKA_100300 and PBANKA_144170) was reduced in *AP2-L*(−) parasites. Because of their structures, these two proteins could be secreted through the plasma membrane of the parasite into the vacuolar space and then transported through the PV membrane to the cytoplasm of the hepatocyte, where they could modulate the functions of host molecules. If these proteins are transported to the host cell, they might be presented to the host immune system as antigens and serve as targets for a protective immune response. Further investigation of AP2-L target genes might provide candidate LS antigens and clues to understanding host-parasite interactions during malarial infection of hepatocytes.

## Materials and Methods

### Ethics Statement

This study was carried out in strict accordance with the recommendations in the Guide for the Care and Use of Laboratory Animals of the National Institutes of Health. The protocol was approved by the Committee on the Ethics of Animal Experiments of the Mie University (Permit Number:23–29). All efforts were made to minimize animal suffering during the course of these studies.

### Parasite Preparations

*Anopheles stephensi* mosquitoes were infected by feeding on mice (6- to 10-wk-old female BALB/c mice, Japan SLC, Inc., Hamamatsu, Japan) infected with the *P. berghei* ANKA strain and maintained at 20°C in a fumed chamber. Oocyst sporozoites and salivary gland sporozoites were harvested from the mosquitoes 14 days and 24 days after their infective blood meal, respectively. LS parasites were cultured as described previously [20]. Briefly, salivary gland sporozoites were added to HepG2 cells (Riken Cell Bank, Tsukuba, Japan) seeded on an 8-well chamber slide, incubated for 30 min at 37°C for invasion, and then cultured at 37°C in 5% CO_2_ in medium containing 3 mg ml^−1^ glucose_._ The medium was changed twice per day.

### Preparation of Antibodies Against UIS4

Antibodies against UIS4 were prepared as follows. A DNA fragment encoding amino acid residues 79–187 of *P. berghei* UIS4 was amplified by PCR by the primer pair 5′-CGGGATCCGAAGTTCGAGAAAAATTTAGAATT-3′ and 5′-CGGCTCGAGTCATTCGTCCTTATGTTCATCTGTAAA-3′ using *P. berghei* genomic DNA as a template. This fragment was digested with *Bam* HI and *Xho* I and then subcloned into the *Escherichia coli* expression vector pGEX 6P-1 (Amersham Pharmacia Biotech, Piscataway, NJ). Recombinant protein was produced in the BL21 *E. coli* strain as a glutathione S-transferase (GST) fusion protein, purified with a glutathione column, and used to immunize rabbits. Antibodies against GST were removed from the antisera by incubation with GST-conjugated Sepharose. Specific antibodies were affinity-purified using an NHS-activated High Trap column (Amersham Pharmacia Biotech) linked with the GST-fused recombinant protein.

### Targeted Disruption of *P. berghei* Genes

Targeted disruption of *P. berghei* genes was carried out in essentially the same procedure as described previously [12]. The PCR primer pairs used to prepare the targeting constructs and the Southern hybridization probes are listed in Table S3.

### Preparation of Fluorescent Protein-tagged AP2-L–expressing Parasites

GFP-tagged AP2-L-expressing parasites were prepared as described previously [12]. To prepare the targeted insertion construct (Figure 3A), a DNA fragment containing the 3′ part of the *AP2-L* coding region was amplified by PCR using genomic DNA as a template and inserted into the *Xho*I/*Nhe*I site of the construct in frame with the GFP gene. The downstream region of the *AP2-L* gene was also amplified by PCR and inserted into the *BamH*I/*Not*I site. The plasmids containing the construct were digested with *Xho*I and *Not*I and then used for transfection. The PCR primer pairs used to prepare the construct and the Southern hybridization probe are listed in Table S3. To monitor AP2-L expression in the LS, mCherry-tagged AP2-L-expressing parasites were prepared as described above but with the targeted insertion construct containing the mCherry gene and 507cl1 parasites, which constitutively express GFP.

### Sporozoite Invasion Assays

For the *in vitro* assays, salivary gland sporozoites were harvested 24 days after an infective blood meal. Cell-traversal assays were performed essentially as described [20]. Double staining assays were performed using 5×10^4^ sporozoites per well essentially as described [13], except that the chamber slides were centrifuged at 140 ×*g* for 2 min at RT after sporozoite inoculation. For PV formation assay, salivary gland sporozoites were inoculated into a culture of HepG2 cells seeded on an 8-well chamber slide. After incubation for 5 or 7 h at 37°C, the slide was fixed in 4% paraformaldehyde for 10 min followed by methanol for 10 min. The newly formed PVM surrounding the sporozoites was stained with a primary antibody against UIS4 (rabbit polyclonal antibody), followed by a secondary antibody conjugated to FITC. After washing with PBS and fixation in methanol for 10 min, sporozoites were stained with a primary antibody against CSP (rat polyclonal antibody), followed by a secondary antibody conjugated with Cy3. To assess liver infection ability, the harvested salivary gland sporozoites were injected into a rat tail vein, and parasitemia was examined using Giemsa staining of a blood smear.

### LS Parasite Culturing and Immunocytochemistry

LS parasites were cultured using HepG2 cells as described above. The cultures were harvested at different time points and fixed in 4% paraformaldehyde for 10 min and then in methanol for 10 min. LS parasites were stained with a primary antibody against the CSP repeat region [20] followed by a secondary antibody conjugated with FITC. Nuclei were stained by the addition of 4,6 diamidino-2-phenylindol (DAPI, 0.02 µg ml^−1^ final concentration) to the secondary antibody solution. At each time point, the largest and smallest diameter of each LS parasite was measured using Aqua Cosmos software (Hamamatsu Photonics, Hamamatsu, Japan), and the average was used to compare the development of LS parasites.

### DNA Microarray

Custom microarrays were designed by e-array, a web-based application (https://earray.chem.agilent.com/earray/), based on the gene annotation of the *P. berghei* genome published in PlasmoDB (http://plasmodb.org). An array in the format of four wells per slide, with four probes attributed to each gene, was used for the analysis of the LS parasites. An array in the format of eight wells per slide, with three probes attributed to each gene, was used for the analysis of the sporozoite stages. Total RNA extraction and the subsequent microarray analyses were performed as described previously [12]. For the analysis of gene expression in the LS parasites, ten and seven biologically independent samples were prepared from wild-type and *AP2-L*(−) parasites, respectively. For the analysis of the sporozoite stages, five biologically independent samples were prepared from each genotype. The obtained data were analyzed with Genespring software (Agilent Technologies). Briefly, all expression data on a chip were normalized to the 60^th^ percentile of the measurements taken from all values on that chip. After normalization, the expression values for each probe set were filtered for raw signal (>100). The genes satisfying this filter were further analyzed for differences between wild-type and *AP2-L*(−) parasites using ANOVA (analysis of variance) with Benjamini-Hochberg multiple testing correction (P0.05). Finally, the genes whose expression decreased least threefold in all attributed probes as compared with wild-type parasites were evaluated as “reduced” genes. From the selected genes, those belonging to multigene families, such as the bir gene family, were excluded. All microarray data were submitted to the Gene Expression Omnibus (GEO) under Accession No. GSE34878.

### RT-PCR

Fifty nanograms of total RNA was treated with DNase and reverse-transcribed with random primers using a PrimeScript® RT reagent Kit with gDNA Eraser (Takara Bio Inc., Otsu, Japan) according to the manufacturer’s protocol. Quantitative PCR was performed as described previously [12]. The PCR primer pairs are listed in Table S3.

### Computational Analysis

The AP2-L binding motifs in the upstream regions of the candidate target genes were investigated with essentially the same statistical methods as reported in our previous papers [11], [12]. In brief, the number of all possible five- to eight-base sequences was counted in the upstream regions of all *P. berghei* genes. Student’s t-test was then carried out for each sequence between candidate target genes (11 genes) and all other *P. berghei* genes, and the sequences that appeared in the former group in significantly high frequencies were investigated.

## Supporting Information

Figure S1 An amino acid sequence near the carboxyl-terminal end of *P. berghei* AP2-L is conserved in other apicomplexan AP2-family proteins. A. Sequence comparison between *P. berghei* AP2-L and its paralogs. B. Sequence comparison between *P. berghei* AP2-L and related proteins in other apicomplexan parasites.
(TIF)

Figure S2 Expression profile of AP2-L in intra-erythrocytic stages. The expression of GFP-tagged AP2-L in *AP2-L::GFP* parasites was observed with fluorescence microscopy. Nuclei were stained with Hoechst stain. The scale bars represent 10 µm.
(TIF)

Figure S3 AP2-L is localized in the nucleus of trophozoites and salivary gland sporozoites. The expression of mCherry-tagged AP2-L in *AP2-L::mCherry* parasites was observed with fluorescence microscopy. Nuclei were stained with Hoechst stain. A trophozoite is indicated by an arrowhead. A schizont is observed in the upper left corner. The scale bar represents 10 µm.
(TIF)

Figure S4 AP2-L disappears in mature liver schizonts. *AP2-L::mCherry* LS parasites were cultured for 48 h in HepG2 cells. After fixation with 4% paraformaldehyde for 10 min and nuclear staining with DAPI, the parasites were examined by fluorescence microscopy. AP2-L was observed only in immature, small LS parasites (indicated by arrows). The scale bar represents 10 µm.
(TIF)

Figure S5 Schematic diagram of the targeted knockout of *P. berghei SLARP* by double cross-over homologous recombination. Targeted knockout of the SLARP gene was performed by essentially the same procedure as for the AP2-L gene. The result of a Southern blot analysis of wild-type (WT) and a knockout population (*SLARP*(−)−1) is shown at the bottom of the figure.
(TIF)

Figure S6 *AP2-L* expression is decreased in *SLARP*(−) salivary gland sporozoites. RT-PCR analysis of *AP2-L* in wild-type (WT) and *SLARP*(−) salivary gland sporozoites 24 days after an infective blood meal. The data (mean ± SEM of four measurements) were normalized to the transcript levels of *CSP*.
(TIF)

Figure S7 Expression of *UIS3, UIS4* and *LISP1* in *AP2-L*(−) LS parasites. RT-PCR analysis of *UIS3*, *UIS4* and *LISP1* was performed in cultured wild-type (WT) and *SLARP*(−) LS parasites at 24 hpi. The data (mean ± SEM of three measurements) were normalized to the transcript levels *of the HSP70* gene (PBANKA_091440).
(TIF)

Figure S8 Expression of *UIS3* and *UIS4* in *AP2-L*(−) salivary gland sporozoites. RT-PCR analysis of *UIS3* and *UIS4* in wild-type (WT) and *AP2-L*(−) salivary gland sporozoites 24 days after an infective blood meal. The data (mean ± SEM of four measurements) were normalized to the transcript levels of *CSP*.
(TIF)

Table S1 *AP2-L*(−) parasites infect mosquitoes normally. a. Twenty mosquitoes were dissected 14 days after an infective blood meal, and the number of parasites per mosquito was calculated (standard error in parentheses). b. Twenty mosquitoes were dissected 24 days after an infective blood meal, and the number of parasites per mosquito was calculated (standard error in parentheses).
(DOC)

Table S2 The fusion of GFP or mCherry to AP2-L does not affect the infective ability of P. *berghei* parasites. a. Salivary gland sporozoites were collected 24 d after an infective blood meal. b. The average prepatent time of infected rats. Parasitemia was checked at one-day intervals starting two days after the challenge.
(DOC)

Table S3 Primers used in this study.
(DOC)
