# Supplementary figures and images for: Identification of an AP2-family Protein That Is Critical for Malaria Liver Stage Development
Source: PLoS One. 2012 Nov 7;7(11):e47557. doi: 10.1371/journal.pone.0047557 (PMC3492389; doi:10.1371/journal.pone.0047557)

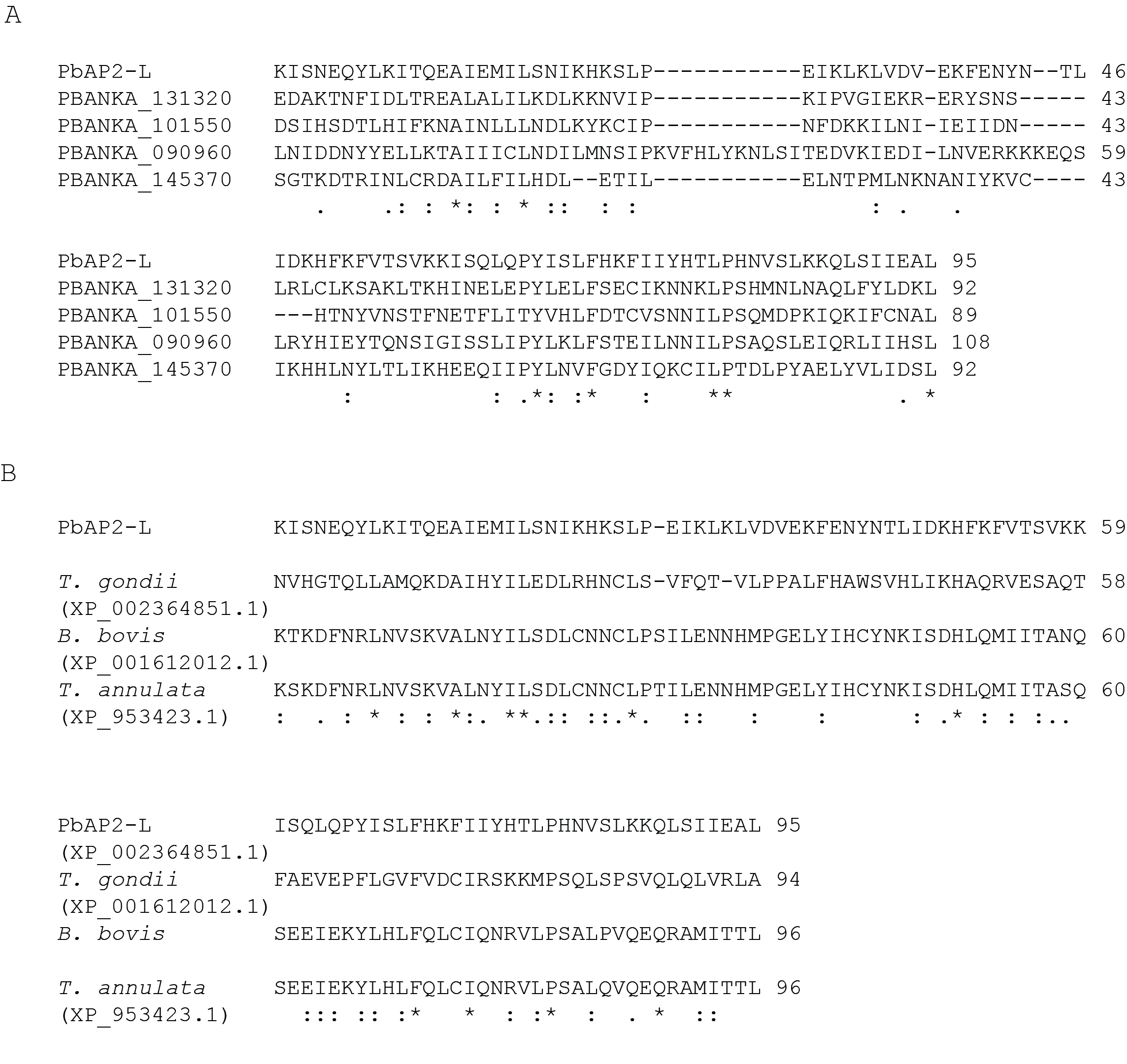

Supplement: Figure S1 — An amino acid sequence near the carboxyl-terminal end of P. berghei AP2-L is conserved in other apicomplexan AP2-family proteins. A. Sequence comparison between P. berghei AP2-L and its paralogs. B. Sequence comparison between P. berghei AP2-L and related proteins in other apicomplexan parasites. (TIF) [file pone.0047557.s001.tif]

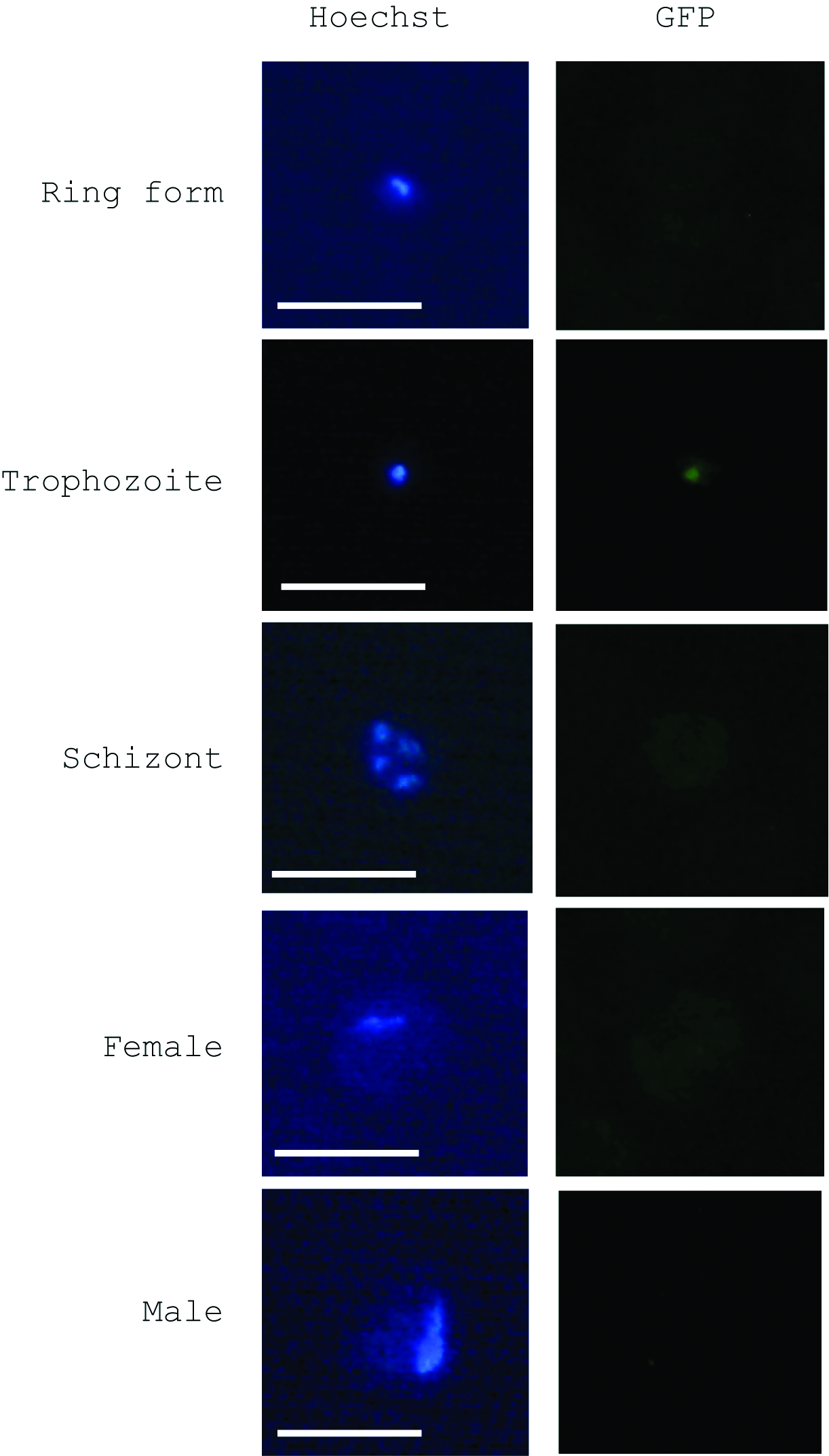

Supplement: Figure S2 — Expression profile of AP2-L in intra-erythrocytic stages. The expression of GFP-tagged AP2-L in AP2-L::GFP parasites was observed with fluorescence microscopy. Nuclei were stained with Hoechst stain. The scale bars represent 10 µm. (TIF) [file pone.0047557.s002.tif]

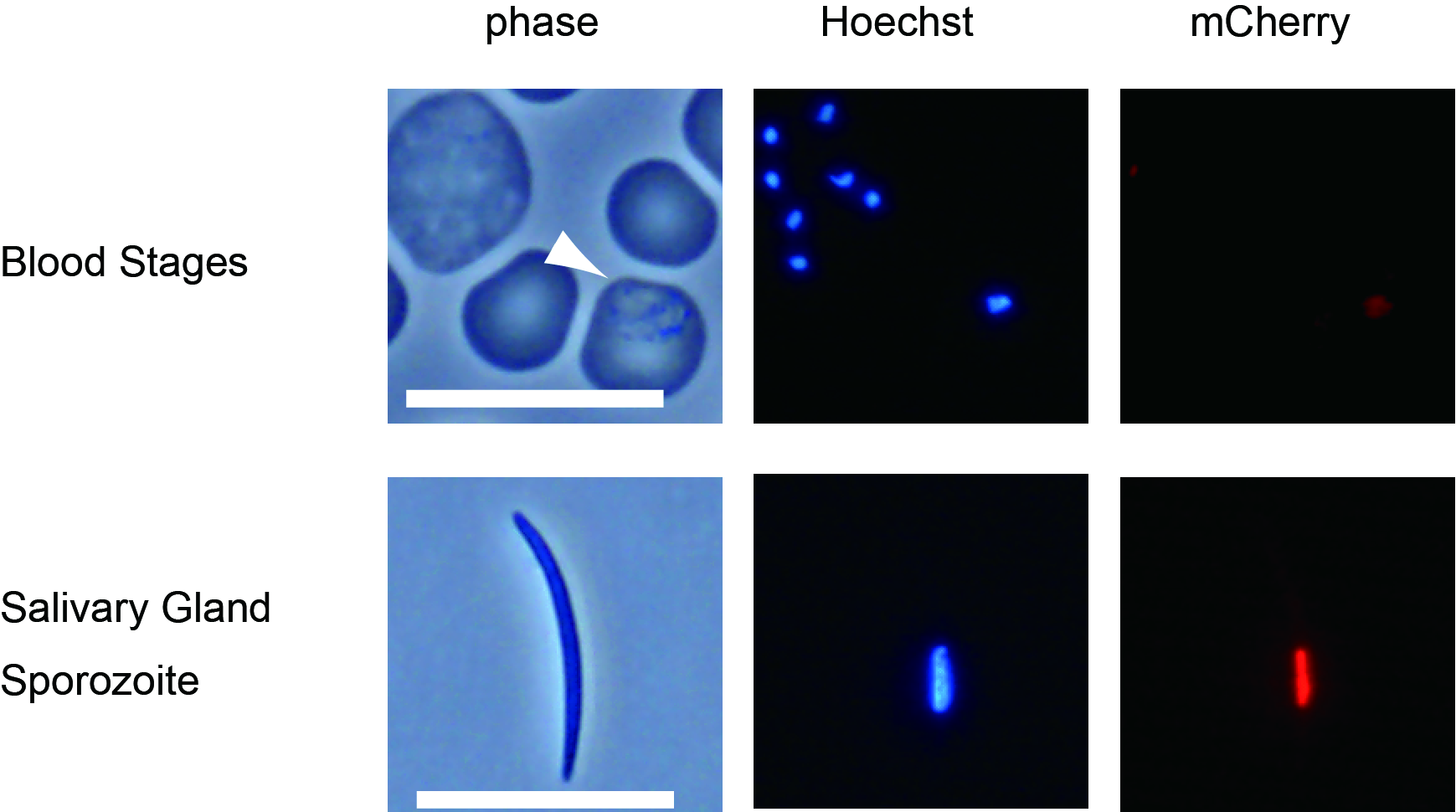

Supplement: Figure S3 — AP2-L is localized in the nucleus of trophozoites and salivary gland sporozoites. The expression of mCherry-tagged AP2-L in AP2-L::mCherry parasites was observed with fluorescence microscopy. Nuclei were stained with Hoechst stain. A trophozoite is indicated by an arrowhead. A schizont is observed in the upper left corner. The scale bar represents 10 µm. (TIF) [file pone.0047557.s003.tif]

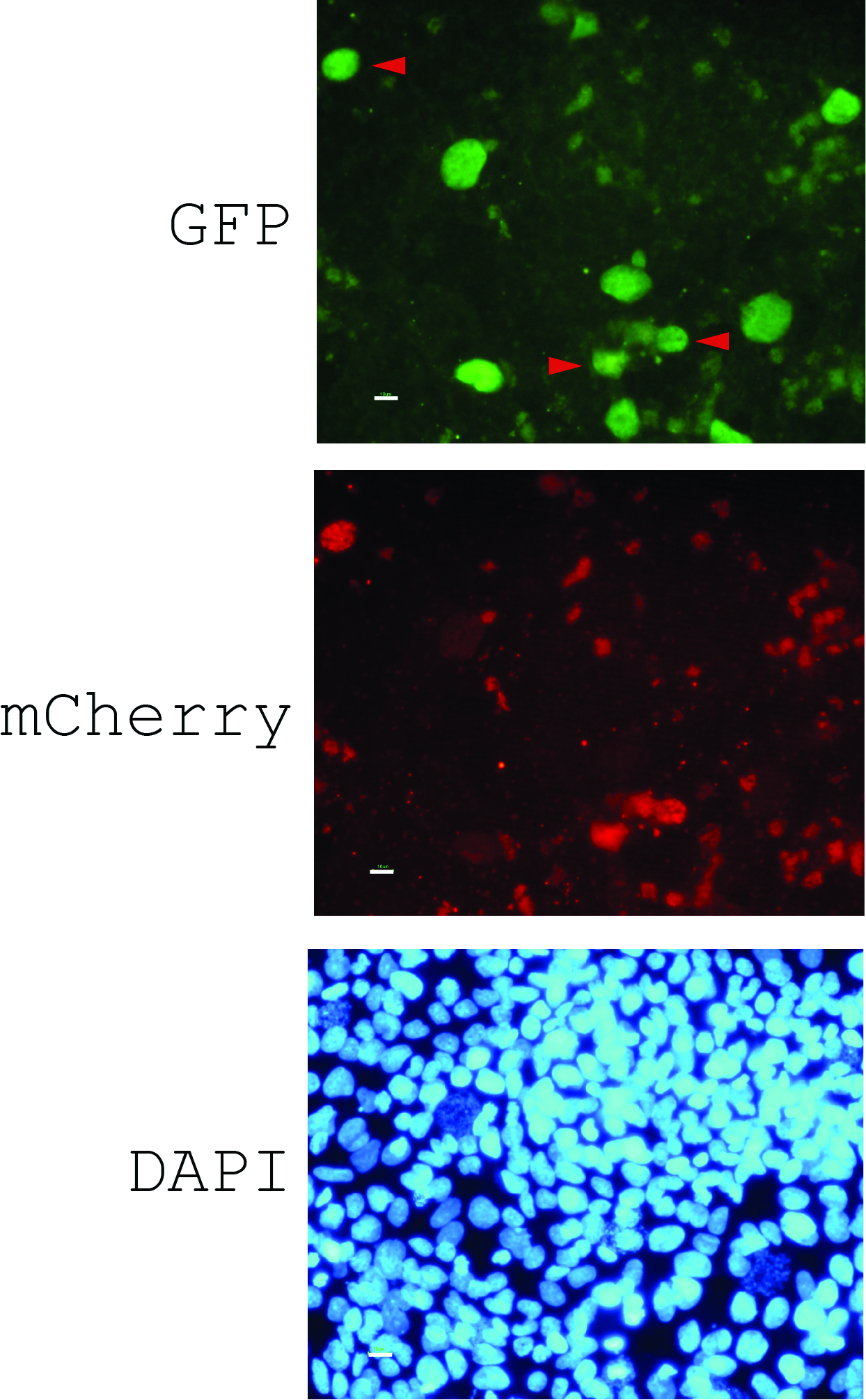

Supplement: Figure S4 — AP2-L disappears in mature liver schizonts. AP2-L::mCherry LS parasites were cultured for 48 h in HepG2 cells. After fixation with 4% paraformaldehyde for 10 min and nuclear staining with DAPI, the parasites were examined by fluorescence microscopy. AP2-L was observed only in immature, small LS parasites (indicated by arrows). The scale bar represents 10 µm. (TIF) [file pone.0047557.s004.tif]

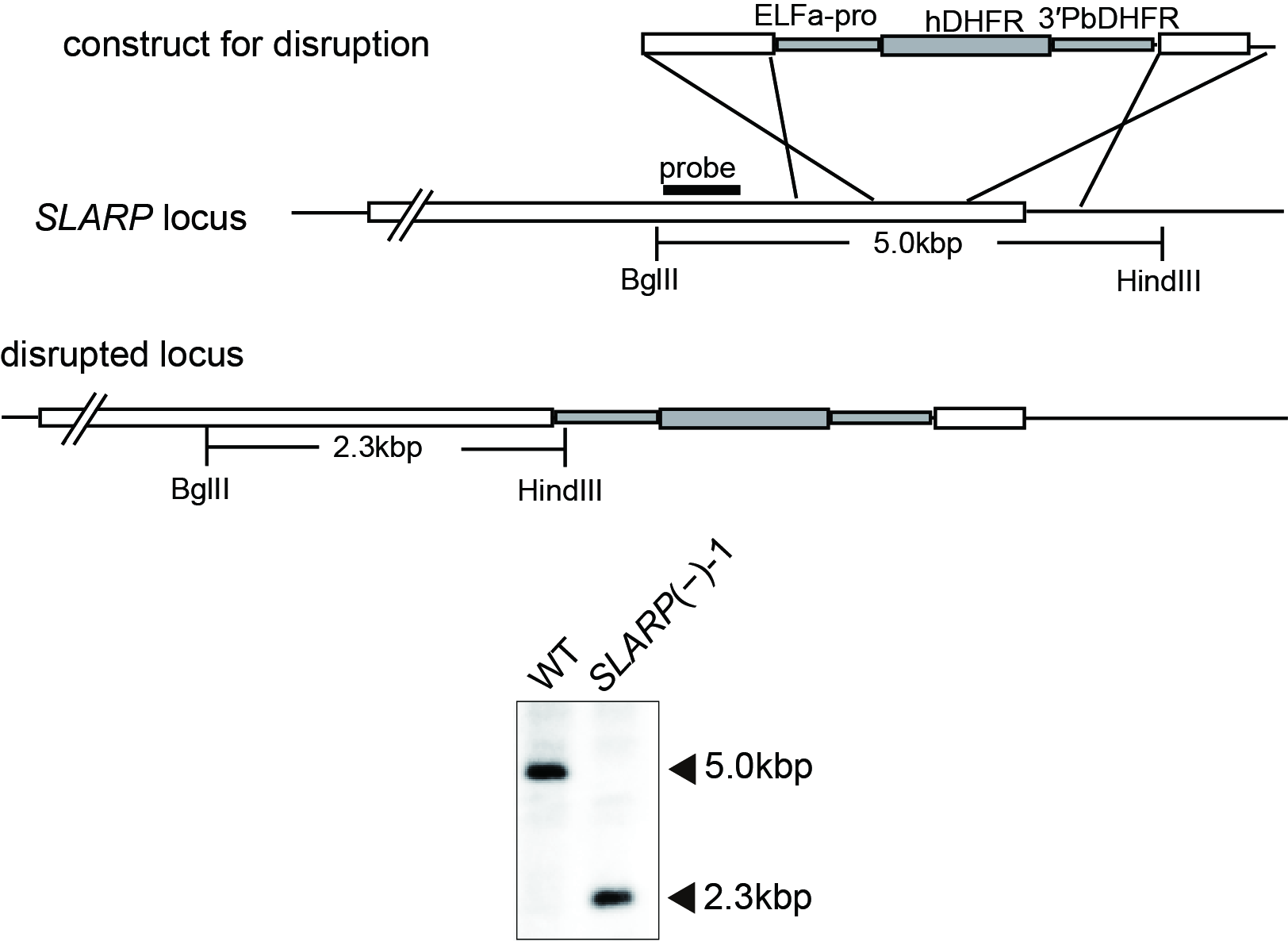

Supplement: Figure S5 — Schematic diagram of the targeted knockout of P. berghei SLARP by double cross-over homologous recombination. Targeted knockout of the SLARP gene was performed by essentially the same procedure as for the AP2-L gene. The result of a Southern blot analysis of wild-type (WT) and a knockout population (SLARP(−)−1) is shown at the bottom of the figure. (TIF) [file pone.0047557.s005.tif]

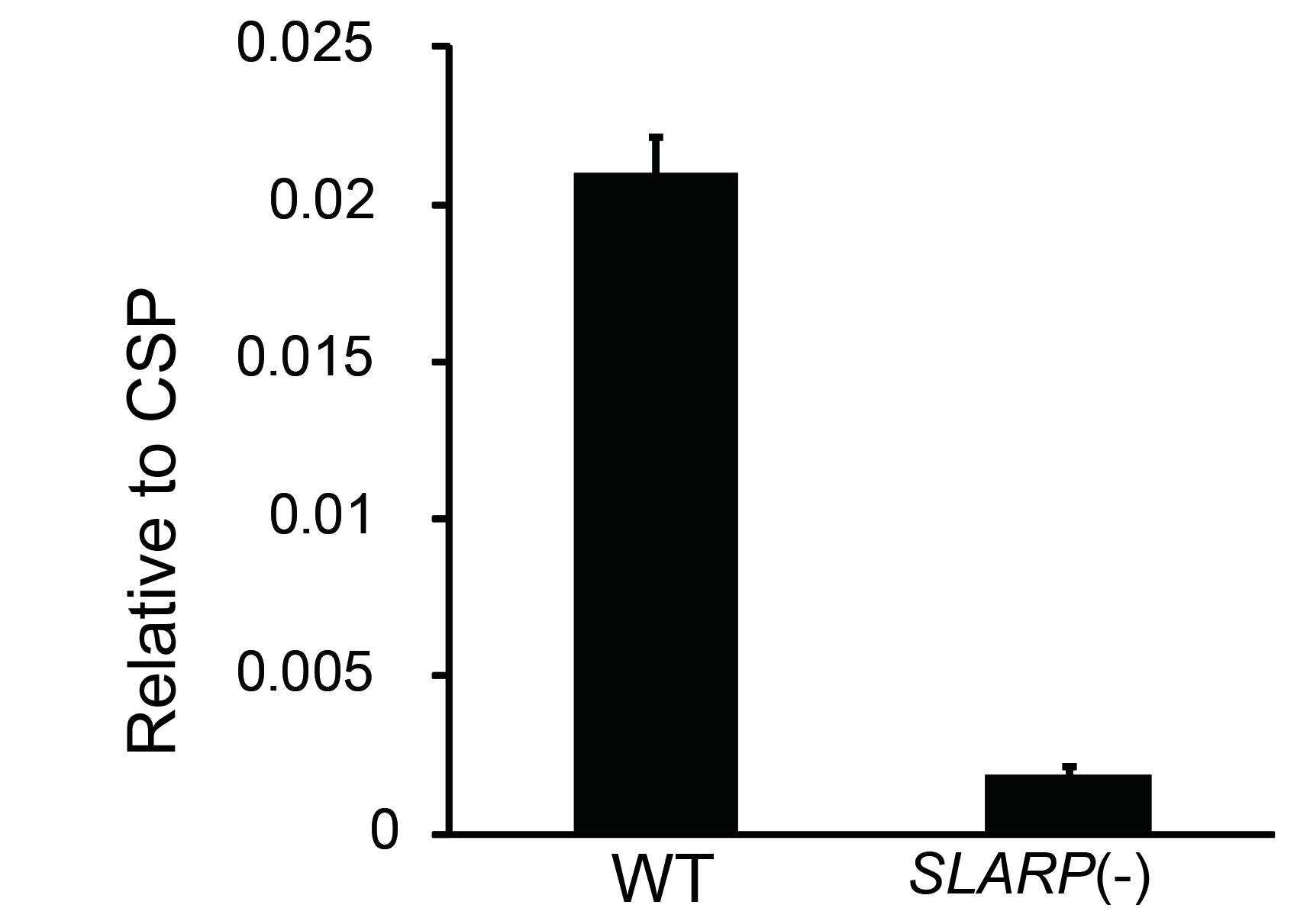

Supplement: Figure S6 — AP2-L expression is decreased in SLARP(−) salivary gland sporozoites. RT-PCR analysis of AP2-L in wild-type (WT) and SLARP(−) salivary gland sporozoites 24 days after an infective blood meal. The data (mean ± SEM of four measurements) were normalized to the transcript levels of CSP. (TIF) [file pone.0047557.s006.tif]

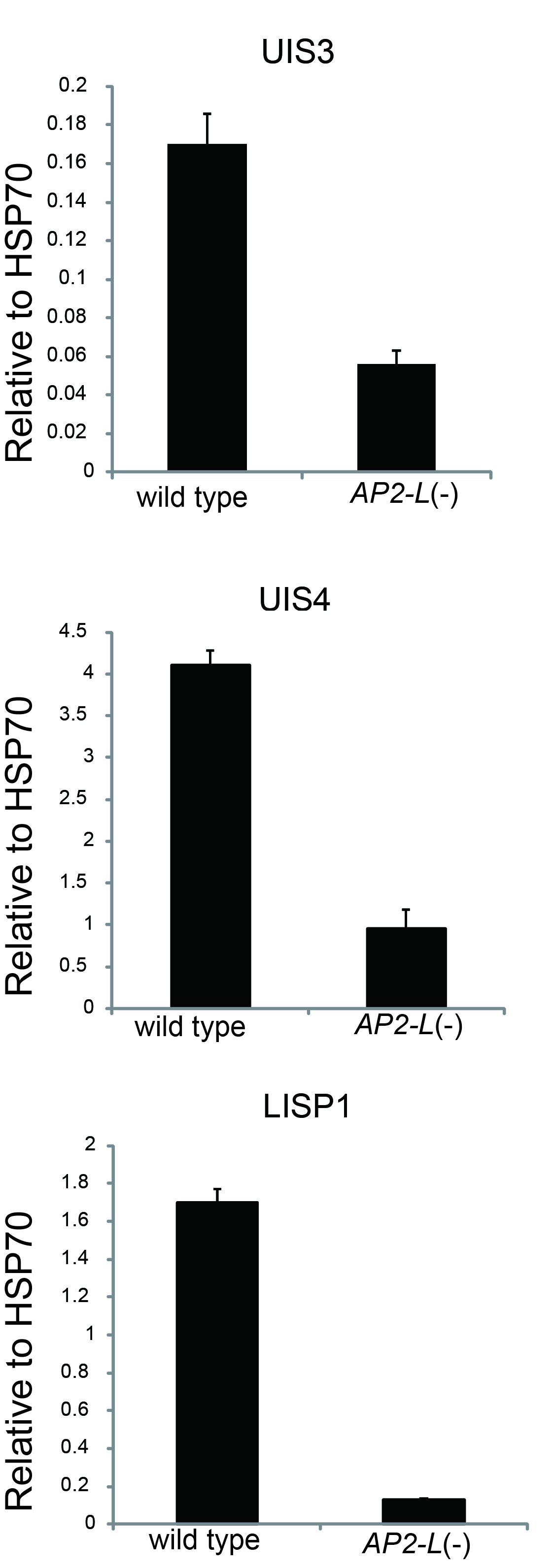

Supplement: Figure S7 — Expression of UIS3, UIS4 and LISP1 in AP2-L(−) LS parasites. RT-PCR analysis of UIS3, UIS4 and LISP1 was performed in cultured wild-type (WT) and SLARP(−) LS parasites at 24 hpi. The data (mean ± SEM of three measurements) were normalized to the transcript levels of the HSP70 gene (PBANKA_091440). (TIF) [file pone.0047557.s007.tif]

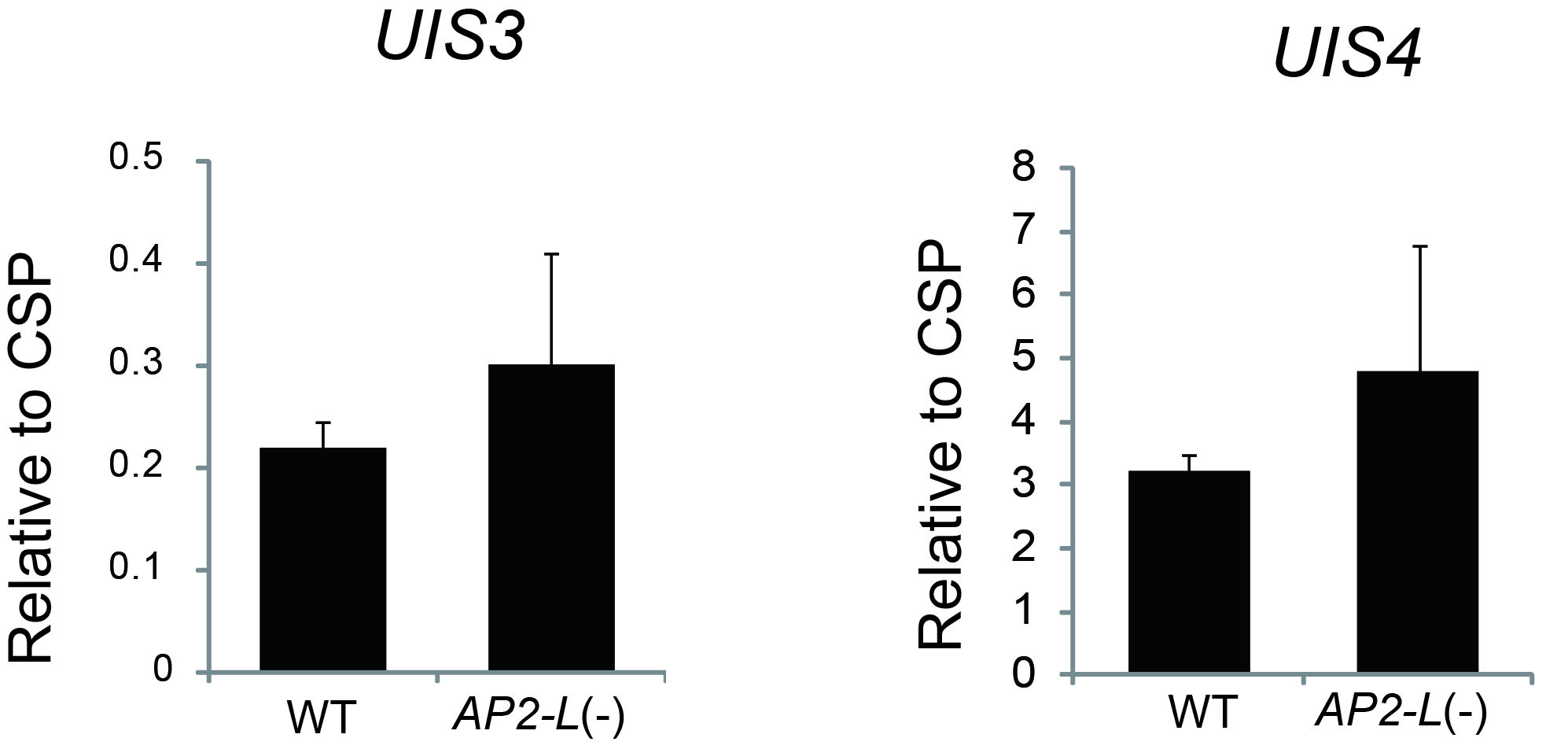

Supplement: Figure S8 — Expression of UIS3 and UIS4 in AP2-L(−) salivary gland sporozoites. RT-PCR analysis of UIS3 and UIS4 in wild-type (WT) and AP2-L(−) salivary gland sporozoites 24 days after an infective blood meal. The data (mean ± SEM of four measurements) were normalized to the transcript levels of CSP. (TIF) [file pone.0047557.s008.tif]
