# Supplementary material for: Identification of an AP2-family Protein That Is Critical for Malaria Liver Stage Development
Source: PLoS One. 2012 Nov 7;7(11):e47557. doi: 10.1371/journal.pone.0047557 (PMC3492389; doi:10.1371/journal.pone.0047557)
Supplement: Table S1 — AP2-L(−) parasites infect mosquitoes normally. a. Twenty mosquitoes were dissected 14 days after an infective blood meal, and the number of parasites per mosquito was calculated (standard error in parentheses). b. Twenty mosquitoes were dissected 24 days after an infective blood meal, and the number of parasites per mosquito was calculated (standard error in parentheses). (DOC) [file pone.0047557.s009.doc]

| Genotype | Number of oocysts per mosquito^a^ | Number of oocyst sporozoites per mosquito^a^ | Number of salivary gland sporozoites per mosquito^b^ |
| --- | --- | --- | --- |
| Wild-type | 96.7 (27.0) | 26,000 (5690) | 10,500 (1420) |
| *AP2-L*(­) 1 | 58.4 (12.6) | 20,700 (4100) | 11,400 (2030) |
| *AP2-L*(­) 2 | 70.3 (21.1) | 23,000 (5370) | 12,600 (2880) |
