# Supplementary material for: Identification of an AP2-family Protein That Is Critical for Malaria Liver Stage Development
Source: PLoS One. 2012 Nov 7;7(11):e47557. doi: 10.1371/journal.pone.0047557 (PMC3492389; doi:10.1371/journal.pone.0047557)
Supplement: Table S2 — The fusion of GFP or mCherry to AP2-L does not affect the infective ability of P. berghei parasites. a. Salivary gland sporozoites were collected 24 d after an infective blood meal. b. The average prepatent time of infected rats. Parasitemia was checked at one-day intervals starting two days after the challenge. (DOC) [file pone.0047557.s010.doc]

| Parasite | Number of injected sporozoites^a^ | Number of infected rats /injected | Prepatent time (days)^b^ |
| --- | --- | --- | --- |
| *507cl1* | 30,000 | 4/4 | 3.0 |
| *AP2-L::GFP* | 30,000 | 4/4 | 3.0 |
| *AP2-L::mCherry* | 30,000 | 4/4 | 3.0 |
