# Supplementary material for: Identification of an AP2-family Protein That Is Critical for Malaria Liver Stage Development
Source: PLoS One. 2012 Nov 7;7(11):e47557. doi: 10.1371/journal.pone.0047557 (PMC3492389; doi:10.1371/journal.pone.0047557)
Supplement: Table S3 — Primers used in this study. (DOC) [file pone.0047557.s011.doc]

| RT-qPCR | AP2-L F | GTTGGTGGTGTTTGCTTTGA |
| --- | --- | --- |
|  | AP2-L R | TCCATCGATTTCTTGGCTGT |
|  | CS F | ATCCAAGCCCAAAGGAACTT |
|  | CS R | GGAGCATCGGCAAGTAATCT |
|  | UIS4 F | CCATACCCAGAAAATTCACCA |
|  | UIS4 R | TGCTTCTTCAGTGGGGCTAT |
|  | UIS3 F | CATCAGGACTGGTGGCAAGT |
|  | UIS3 R | GCTTGGCTTTTCTCCATCAG |
|  | HSP70 F | TGCAGCAGATAATCAAACTC |
|  | HSP70 R | ACTTCAATTTGTGGAACACC |
|  | rat GAPDH F | gtggacctcatggcctacat |
|  | rat GAPDH R | tgtgagggagatgctcagtg |
|  | berghei 18S F | GGGGATTGGTTTTGACGTTTTTGCG |
|  | berghei 18S R | AAGCATTAAATAAAGCGAATACATCCTTAT |
| *AP2-L*(-) parasites | AP2-L-1 | GATGATGAAAATGAAGAGAATGAGG |
|  | AP2-L-2 | CTCATCTACAAGCATCgtcgacAATAGTTAGATTCATACGACTCGC |
|  | AP2-L-3 | CCTTCAATTTCGgatccactagGCCGCACACGCTCACATATGC |
|  | AP2-L-4 | TATCAATAAAGCTGATATTTCGTTACG |
|  | Southern probe-F | GAGGGATCACGTGTGCTATAC |
|  | Southern probe-R | GCTATTTAGGATAATTGATGCACTATAG |
| *GFP* (*mCherry*)  *::AP2-L* parasites | AP2-L-GFP 5F | aaactcgagGAAGCAAGAGATAGAGCTATTTATTGC |
|  | AP2-L-GFP 5R | aaagctagcATCAATTTTATGGTTAACGTCGCCAG |
|  | AP2-L-GFP 3F | aaaggatccGTGAAACGGTATAAAGGGGTGAAACAC |
|  | AP2-L-GFP 3R | aaagcggccgcGCTTGTCCTGAGAGATATAAGGATCC |
|  | Southern probe-F | aaactcgagGAAGCAAGAGATAGAGCTATTTATTGC |
|  | Southern probe-R | aaagctagcATCAATTTTATGGTTAACGTCGCCAG |
| *SLARP*(-) parasites | SLARP-1 | CCATTGAAGGTACCAGCATTATCA |
|  | SLARP -2 | CTCATCTACAAGCATCgtcgacGGGCAAGGCTCGGTGTCTTC |
|  | SLARP-3 | CCTTCAATTTCGgatccactagCTTCATAAGCATACACATTCGTGTG |
|  | SLARP-4 | GTTTTTTGGTCATTTATGTGCATG |
|  | Southern probe-F | ATTTAGCATGGGAATTCATAACAATAACG |
|  | Southern probe-R | CATTGGGACCTTTAATATTATTATAATTGC |
